# Aliased Flow Signal Planimetry by Cardiovascular Magnetic Resonance Imaging for Grading Aortic Stenosis Severity: A Prospective Pilot Study

**DOI:** 10.3389/fcvm.2021.752340

**Published:** 2021-10-18

**Authors:** Cesare Mantini, Mohammed Y. Khanji, Emilia D'Ugo, Marzia Olivieri, Cristiano Giovanni Caputi, Gabriella Bufano, Domenico Mastrodicasa, Darien Calvo Garcia, Domenico Rotondo, Matteo Candeloro, Claudio Tana, Filippo Cademartiri, Adrian Ionescu, Massimo Caulo, Sabina Gallina, Fabrizio Ricci

**Affiliations:** ^1^Department of Neuroscience, Imaging and Clinical Sciences, “G. d'Annunzio” University of Chieti-Pescara, Chieti, Italy; ^2^Newham University Hospital, Barts Health NHS Trust, London, United Kingdom; ^3^Barts Heart Centre, Barts Health NHS Trust, London, United Kingdom; ^4^National Institute for Health Research (NIHR) Barts Biomedical Research Centre, William Harvey Research Institute, Queen Mary University of London, London, United Kingdom; ^5^Azienda Sanitaria Locale (ASL) 2 Lanciano Vasto Chieti, Regione Abruzzo, Italy; ^6^Department of Radiology, Stanford University, Stanford, CA, United States; ^7^Department of Innovative Technologies in Medicine and Dentistry, “G. d'Annunzio” University of Chieti-Pescara, Chieti, Italy; ^8^SDN Istituto di Ricovero e Cura a Carattere Scientifico (IRCCS), Naples, Italy; ^9^Department of Cardiology, Morriston Cardiac Regional Centre, Swansea Bay Health Board, Swansea, United Kingdom; ^10^Department of Clinical Sciences, Lund University, Malmö, Sweden; ^11^Casa di Cura Villa Serena, Città Sant'Angelo, Pescara, Italy

**Keywords:** CMR, echocardiography, aortic stenosis (AS), valvular heart disease, phase contrast (PC), aliasing analysis

## Abstract

**Objectives:** Transthoracic echocardiography (TTE) is the standard technique for assessing aortic stenosis (AS), with effective orifice area (EOA) recommended for grading severity. EOA is operator-dependent, influenced by a number of pitfalls and requires multiple measurements introducing independent and random sources of error. We tested the diagnostic accuracy and precision of aliased orifice area planimetry (AOA_cmr_), a new, simple, non-invasive technique for grading of AS severity by low-VENC phase-contrast cardiovascular magnetic resonance (CMR) imaging.

**Methods:** Twenty-two consecutive patients with mild, moderate, or severe AS and six age- and sex-matched healthy controls had TTE and CMR examinations on the same day. We performed analysis of agreement and correlation among (i) AOA_cmr_; (ii) geometric orifice area (GOA_cmr_) by direct CMR planimetry; (iii) EOA_echo_ by TTE-continuity equation; and (iv) the “gold standard” multimodality EOA (EOA_hybrid_) obtained by substituting CMR LVOT area into Doppler continuity equation.

**Results:** There was excellent pairwise positive linear correlation among AOA_cmr_, EOA_hybrid_, GOA_cmr_, and EOA_echo_ (*p* < 0.001); AOA_cmr_ had the highest correlation with EOA_hybrid_ (*R*^2^ = 0.985, *p* < 0.001). There was good agreement between methods, with the lowest bias (0.019) for the comparison between AOA_cmr_ and EOA_hybrid_. AOA_cmr_ yielded excellent intra- and inter-rater reliability (intraclass correlation coefficient: 0.997 and 0.998, respectively).

**Conclusions:** Aliased orifice area planimetry by 2D phase contrast imaging is a simple, reproducible, accurate “one-stop shop” CMR method for grading AS, potentially useful when echocardiographic severity assessment is inconclusive or discordant. Larger studies are warranted to confirm and validate these promising preliminary results.

## Introduction

In daily clinical practice, transthoracic echocardiography (TTE) is the primary imaging modality for the initial assessment of suspected aortic stenosis (AS) and for the measurement of effective orifice area (EOA) and geometric orifice area (GOA) of the aortic valve (class I, Level of Evidence B) (1–3). EOA is the key metric in AS and it correlates with survival and need for valve replacement. It is calculated using the continuity equation, with its multiple, independent measurements (aortic jet velocity, left ventricular outflow diameter, and left ventricular outflow velocity), assumptions (circular cross-section of the LVOT, location of the sample volume exactly in the same plane in which the LVOT diameter is measured), and propagating errors (LVOT diameter is divided by 2, squared, and then multiplied by 3.14 and by the LVOT VTI, which magnifies any error considerably) (4–6). Moreover, EOA—and echocardiography in general—is operator-dependent, and its accuracy may be degraded by limited acoustic “windows,” increasingly prevalent in the global obesity epidemic.

Cardiovascular magnetic resonance (CMR) complements echocardiographic assessment of AS—e.g., by allowing planimetric measurement of the aortic valve area in systole (GOA_cmr_)—providing precise information in patients with reduced cardiac output or with conditions influencing the accuracy of flow velocities or pressure gradient measurement by TTE (7, 8). CMR also allows direct measurement of LV stroke volume (without using geometrical assumptions) and of LVOT area, and is more accurate and reproducible than echocardiography (9), but, when compared to Doppler echocardiography, it underestimates flow velocities (6). This has been attributed to intravoxel dephasing, loss of signal, pressure recovery, and its lower temporal resolution compared to Doppler echocardiography (10).

A hybrid approach, in which LVOT area is measured by CMR and the LVOT and aortic velocities are determined using Doppler echocardiography (EOA_hybrid_) (11–14), should overcome the methodological limitations of 2D echocardiography for measurement of LVOT area and of CMR for measurement of velocities and GOA. However, the hybrid approach is time-consuming, not widely available and expensive, but it represents a non-invasive alternative, potentially useful in patients in whom echocardiographic results are inconclusive or conflicting.

Developing a simpler, robust, reproducible and accurate CMR “one-stop shop” method to estimate EOA in patients with aortic stenosis (AS) would thus satisfy a genuine clinical need. The aim of this prospective, observational, cross-sectional study was to (i) test the accuracy and precision of a new, simple, non-invasive CMR technique to measure aortic valve EOA based on aliased orifice area (AOA_cmr_) planimetry by low-VENC phase-contrast CMR imaging; (ii) investigate the relationship and the diagnostic agreement of AOA_cmr_ with valve area planimetry by bSSFP-CMR images (GOA_cmr_), standard EOA obtained with continuity equation by TTE (EOA_echo_), and gold standard hybrid EOA (EOA_hybrid_).

## Materials and Methods

### Study Population

We enrolled consecutive patients referred to our Radiology Unit between March 2018 and June 2018 for suspected valvular heart disease. We stratified the patient population according to AS severity: mild (1.2 cm^2^/m^2^ ≥ indexed EOA > 0.85 cm^2^/m^2^), moderate (0.85 cm^2^/m^2^ ≥ indexed EOA ≥ 0.6 cm^2^/m^2^), or severe AS (indexed EOA < 0.6 cm^2^/m^2^). Each patient underwent TTE and CMR examinations on the same day.

Exclusion criteria were as follows: (i) age <18 years; (ii) left ventricular ejection fraction <50% by CMR; (iii) bicuspid aortic valve; (iv) at least moderate multi-valvular disease; (v) hemodynamic instability; (vi) atrial fibrillation or frequent supraventricular or ventricular premature contractions; and (vii) poor TTE image quality.

The study was conducted according to the criteria set by the Declaration of Helsinki. Patient's informed consent was obtained from all individual participants included in the study. All patients underwent a standard clinical assessment including medical history, physical examination, routine blood tests, transthoracic echocardiogram, and CMR.

### Study Protocol

**Figure 1:**
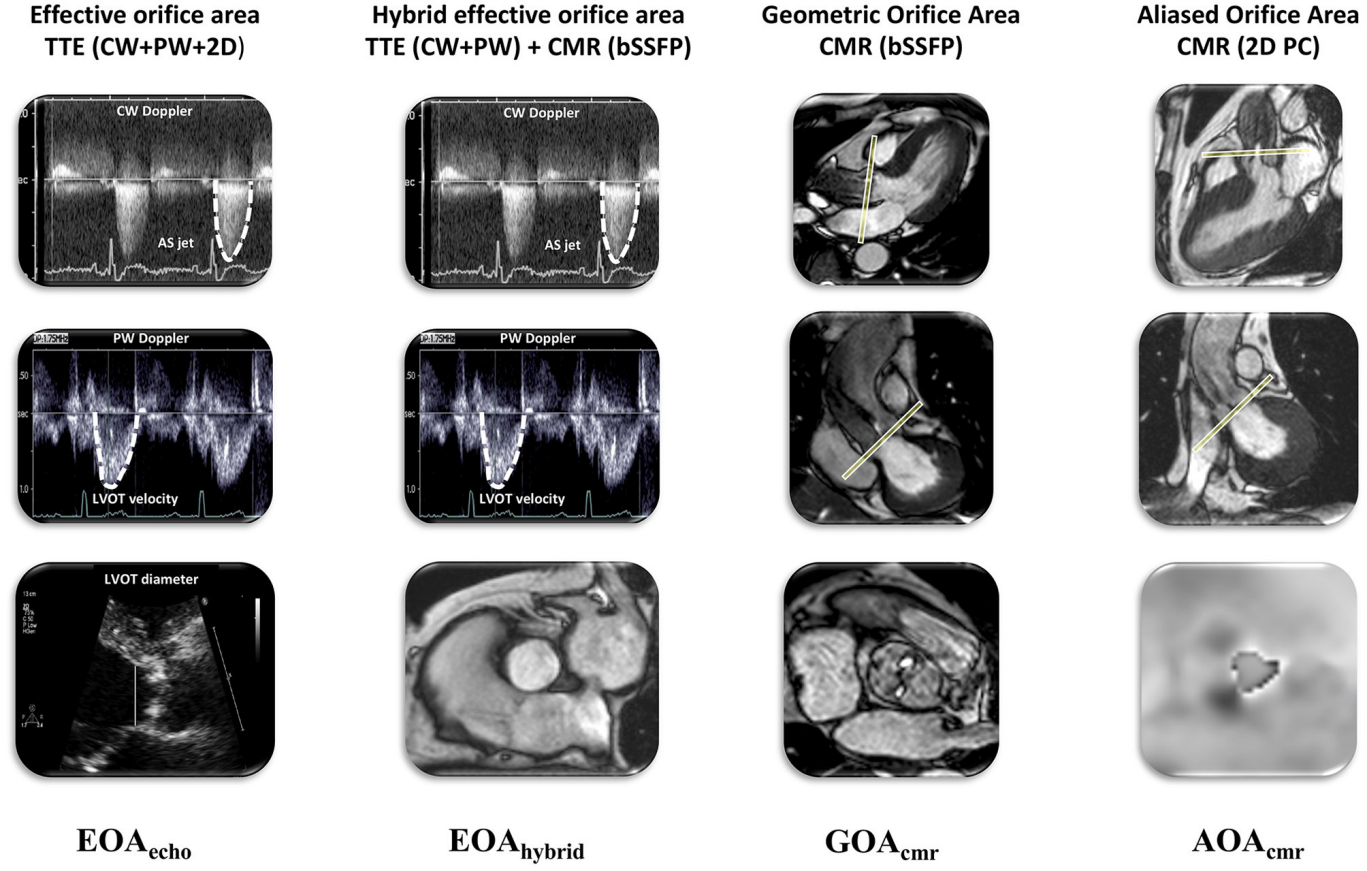
Summary of different methods for non-invasive assessment of aortic valve area and grading of aortic stenosis severity: EOA by TTE, hybrid EOA by TTE and CMR, GOA and EOA by CMR. AOA, aliased orifice area; EOA, effective orifice area; CMR, cardiovascular magnetic resonance; GOA, geometric orifice area; echo, echocardiography; hybrid, combined echo-CMR assessment; TTE, transthoracic echocardiography.

CMR and TTE examinations were performed on the same day by four physicians accredited in each respective imaging modality, without knowledge of the results of the complimentary exam. Imaging and acquisition protocols were those recommended by the respective professional societies (15, 16). We performed the following measurements (Figure 1): (a) effective orifice area (EOA_echo_) by TTE continuity equation; (b) geometric aortic valve area by CMR (GOA_cmr_); (c) hybrid effective orifice area (EOA_hybrid_) as the continuity equation using LVOT area measured by CMR and LVOT and aortic velocities measured by pulsed-wave (PW) and continuous wave (CW) Doppler echocardiography, respectively; and (d) aliased orifice area (AOA_cmr_) planimetry using a low-VENC phase-contrast CMR sequence.

### Transthoracic Echocardiography

All patients had TTE by two accredited cardiologists (S.G. and E.D.) using a commercially available ultrasound system (Esaote My Lab 50 Gold, ESAOTE, Genoa, IT) with 1–5 MHz transducers, according to an established protocol (15). EOA_echo_ was calculated by the continuity equation according to the 2017 EACVI/ASE recommendations (3). TTE image quality was measured by the Image Quality Assessment Tool and poor quality defined by a score of <10 (17).

### Cardiovascular Magnetic Resonance

All patients were examined on Achieva 1.5-T scanner (Philips Medical System, Best, the Netherlands) using a dedicated eight-channel phased-array cardiac synergy coil for signal reception during end-expiratory breath holds. CMR cine assessment with steady-state free-precession sequences (SSFP) provided both morphological and functional data, including left and right ventricular volumes and ejection fraction.

#### GOA_cmr_

**Figure 2:**
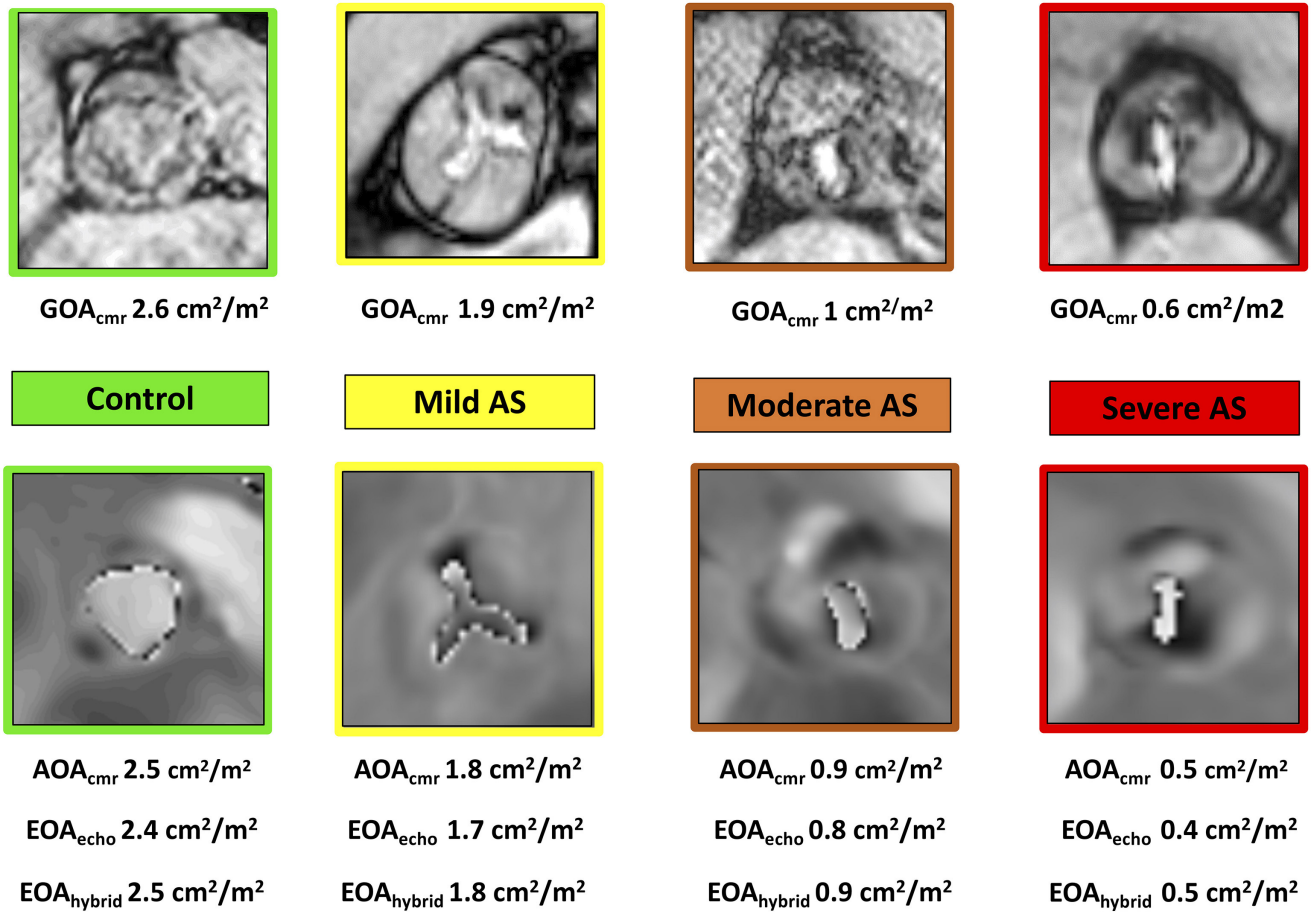
Case examples of normal aortic valve area (green), mild (yellow), moderate (orange), and severe (red) aortic stenosis measured with GOA planimetry (top row) by cine CMR and AOA planimetry by fixed low-VENC phase contrast CMR (bottom row). All measurements have been indexed to body surface area and reported as cm^2^/m^2^. AOA, aliased orifice area; EOA, effective orifice area; CMR, cardiovascular magnetic resonance; GOA, geometric orifice area; echo, echocardiography; hybrid, combined echo-CMR assessment.

GOA_cmr_ using three-chamber and left ventricular outflow tract (LVOT) coronal cine views, six consecutive, parallel cross-sectional bSSFP cine-images from the aortic sino-tubular junction to the LVOT (including the valve tips through-plane) were acquired with retrospective gating (30 phases per cardiac cycle) during multiple breath holds. GOA_cmr_ planimetry was performed by tracing the inner edges of the AV leaflets at maximum systolic opening (Figure 2).

Parameters of aortic valve cross-sectional cine-images included slice thickness of 6 mm, gap of −1 mm, TR/TE of 3.4/1.2 ms, flip angle of 40°, and number of excitations (NEX) = 1, yielding an in-plane spatial resolution of 1.4 × 1.4 mm. Phase-contrast MR imaging parameters were as follows: TR/TE of 4.60–4.92/2.76–3.05 ms, flip angle 15°, 30 phases, pixel spacing 1.32–2.07 mm, slice thickness 10 mm, and an acquisition matrix of 256 × 208.

#### AOA_cmr_

AOA_cmr_ using three-chamber and LVOT coronal cine views, a phase-contrast slice was positioned through the tips of aortic valve leaflets during systole, setting the VENC value to 50 cm/s, with five signal averages and free-breathing acquisition. The VENC was intentionally set low in order to induce aliasing and increase the visual separation between tissues and the flow signal. We traced and measured the surface of the area (AOA_cmr_) occupied by the aliased flow signal at the tips of the AV cusps on the systolic frame with the maximal diameter of the flow signal (Figure 2). This area (AOA_cmr_) would be, on basic hemodynamic principles, equivalent to the effective orifice area (EOA) of the AV. Our proposed new method of tracing the area of the aliased flow signal on phase-velocity maps at the tips of the AV cusps allows, effectively, a direct planimetry measurement of the EOA. This eliminates the “derived” nature of EOA by doing away with the sequential measurements, multiple assumptions, and potential sources of error of the continuity equation-derived EROA.

#### EOA_hybrid_

EOA_hybrid_ we calculated the hybrid effective aortic valve area with a previously reported and validated approach (7-10): EOA_hybrid_ = (VTI_LVOT−ECHO_ × AREA_LVOT−CMR_)/VTI_AV−ECHO._

### Image Analysis

All CMR studies were analyzed off-line using a dedicated semi-automated workstation and post-processing software (MR WorkSpace 2.6.3.2, Philips Medical Systems, Nederland B.V.). All measurements were performed off-line by two independent investigators (FR and CM) blinded to clinical and TTE results. We assessed left and right ventricle volumes and function, analyzed the velocity-encoded images and measured aortic valve planimetry on bSSFP and phase images of PC sequences, and LVOT diameters on bSSFP sequences. Geometric valve area and LVOT area were measured in mid-systole when the valve cusps reached the maximum degree of opening, the transvalvular flow was the widest, and LVOT had the largest diameters.

### Statistical Analysis

Continuous variables were expressed as mean ± standard deviation (SD) and categorical variables as counts and percentages. Categorical variables of patients in different groups were compared using Chi-squared test, and continuous variables were compared among groups using Student's *t*-test for unpaired data. Individual differences among groups were compared *post hoc* using Mann–Whitney *U*-tests. Correlation between EOA_hybrid_ and EOA_echo_, GOA_cmr_, and AOA_cmr_ was evaluated by ordinary least-square linear regression. Agreement between different imaging modalities was assessed by Bland–Altman analysis. To assess inter-observer variability, the measurements of GOA_cmr_ and AOA_cmr_ were repeated in all patients by two blinded observers. To further evaluate intra-observer variability of GOA_cmr_ and AOA_cmr_, sequences were analyzed twice by the two observers. Intra- and interobserver reliability was computed by assessing absolute mean differences and using intraclass correlation coefficient (ICC).

A *p*-value of <0.05 was considered statistically significant. A sample size of 28 patients was calculated considering two observations per patient, with a *p*-value <0.05; statistical power of 80%, expected reliability of 0.80, and acceptable reliability of 0.50.

## Results

Of 37 consecutive patients tested, we finally enrolled 28 individuals (mean age 70 ± 9 years; 14 men), namely, 22 AS patients (9 severe, 7 moderate, and 6 mild) and 6 age- and sex-matched controls. Overall, two patients with poor TTE image quality, four with LVEF <50%, and three with frequent ventricular arrhythmias had to be excluded. Baseline clinical and demographic characteristics of the study population are summarized in Table 1. TTE and CMR findings are reported in Table 2. Heart rate and systolic blood pressure were not statistically different between CMR and echocardiography studies (*p* = 0.58 and *p* = 0.86, respectively).

**Table 1:** Baseline clinical and demographic characteristics of the study population.

**Covariates**	**Aortic stenosis** ***n* = 22**	**Controls** ***n* = 6**	***P*-value**
Age, years	70 ± 9	70 ± 7	NS
Male sex, *n* (%)	19 (68)	4 (66)	NS
BMI, kg/m^2^	28.3 ± 3.8	23.2 ± 2.4	0.006
BSA, m^2^	1.9 ± 0.2	1.8 ± 0.2	NS
SBP, mmHg	133 ± 14	116 ± 9	0.011
HR, bpm	68 ± 10	63 ± 6	NS
Hypertension, *n* (%)	18 (82)	0 (0)	<0.001
Diabetes, *n* (%)	5 (23)	0 (0)	NS
Current smoking, *n* (%)	1 (10)	2 (33)	NS
Dyslipidemia, *n* (%)	10 (45)	0 (0)	0.039
Family history of CAD, *n* (%)	2 (20)	1 (17)	NS
Angina, *n* (%)	5 (18)	0 (0)	NS
Syncope, *n* (%)	2 (17)	0 (0)	NS
Dyspnea, *n* (%)	14 (50)	0 (0)	0.006
Atrial fibrillation, *n* (%)	2 (9)	0 (0)	NS

*AF, atrial fibrillation; BMI, body mass index; BSA, body surface area; CAD, coronary artery disease; HR, heart rate; NS, non-significant (p ≥ 0.05); SBP, systolic blood pressure*.

**Table 2:** Echocardiographic and CMR measurements.

**Covariates**	**Severe AS** **(*n* = 9)**	**Moderate AS** **(*n* = 7)**	**Mild AS** **(*n* = 6)**	**Controls** **(*n* = 6)**
**Echocardiography**				
LVOT_diameter_ (mm)	21.2 ± 1.3	22.3 ± 1.8	21.5 ± 1, 9	21.8 ± 1.9
LVOT_area_ (mm^2^)	354 ± 43	392 ± 61	365 ± 63	377 ± 66
LVOT_VTI_ (cm)	22 ± 5.5	22 ± 4	22 ± 4.8	20.3 ± 6.1
AORTA_VTI_ (cm)	100 ± 9	78 ± 17	53 ± 12	27 ± 5
MG (mmHg)	40 ± 7	27 ± 9.7	19 ± 7.4	3.7 ± 1.2
SVi (ml/m^2^)	42 ± 10	43 ± 8	46 ± 14	41 ± 9
Zva (mmHg/ml/m^2^)	4.2 ± 1.2	3.9 ± 0.7	3.4 ± 0.8	3 ± 0.6
LVET (ms)	331 ± 25	315 ± 29	297 ± 20	307 ± 17
AT (ms)	115 ± 20	101 ± 27	97 ± 16	101 ± 16
TAPSE (mm)	24 ± 4	21 ± 6	24 ± 3	26 ± 5
sPAP (mmHg)	31 ± 7	34 ± 11	30 ± 6	27 ± 6
LAVi (ml/m^2^)	49 ± 21	44 ± 19	27 ± 17	25 ± 12
E/e' (cm/s)	8.9 ± 3.2	9.4 ± 3.9	7 ± 4.5	5.5 ± 0.9
**Cardiovascular magnetic resonance**				
LVEDVi (ml/m^2^)	89 ± 44	90 ± 37	92 ± 23	99 ± 42
LVESVi (ml/m^2^)	46 ± 28	46 ± 35	45 ± 21	49 ± 38
SVi (ml/m^2^)	48 ± 17	43 ± 10	48 ± 5	49 ± 9
LVEF (%)	57 ± 12	53 ± 16	74 ± 18	55 ± 14
LVOT_min_ (mm)	22 ± 1.9	22 ± 1.4	22 ± 1.7	22.1 ± 1.6
LVOT_max_ (mm)	23 ± 2.4	25 ± 2.9	24 ± 2.4	24.1 ± 2.8
LVOT_area_ (mm^2^)	397 ± 67	448 ± 56	421 ± 58	421 ± 60
LVMI (g/m^2^)	79 ± 22	87 ± 19	66 ± 17	71 ± 13
**Aortic valve area measurements**				
EOA_echo_ (cm^2^/m^2^)	0.43 ± 0.12	0.57 ± 0.08	1.2 ± 0.24	1.49 ± 0.11
EOA_hybrid_ (cm^2^/m^2^)	0.48 ± 0.09	0.69 ± 0.06	1.2 ± 0.24	1.67 ± 0.08
AOA_cmr_ (cm^2^/m^2^)	0.47 ± 0.11	0.64 ± 0.08	1.2 ± 0.24	1.68 ± 0.11
GOA_cmr_ (cm^2^/m^2^)	0.58 ± 0.15	0.78 ± 0.21	1.3 ± 0.3	1.77 ± 0.14

*AOA_cmr_, aliased orifice area by cardiovascular magnetic resonance; AT, acceleration time; EOA_echo_, effective orifice area by transthoracic echocardiography; EOA_hybrid_, hybrid effective orifice area; GOA_cmr_, geometric orifice area by cardiovascular magnetic resonance; LAVi, left atrial volume index; LVEDVi, left ventricular end-diastolic volume indexed to body surface area; LVEF, left ventricular ejection fraction; LVESVi, end-systolic volume indexed to body surface area; LVMI, left ventricular mass index; LVOT, left ventricular outflow tract; MG, mean gradient; LVET, left ventricular ejection time; sPAP, systolic pulmonary artery pressure; SVi, stroke volume indexed to body surface area; TAPSE, tricuspid annular plane systolic excursion; VTI, velocity time integral; Zva, valvulo-arterial impedance*.

### Comparison of TTE vs. CMR LVOT Area

The mean values of LVOT_cmr_ area were significantly larger than the LVOT_echo_ both in patients with AS (mild, moderate and severe; *p* < 0.001) and in control group patients (*p* < 0.001) (Table 2).

### Comparison of EOA_echo_, GOA_CMR_, AOA_cmr_, and EOA_hybrid_

**Figure 3:**
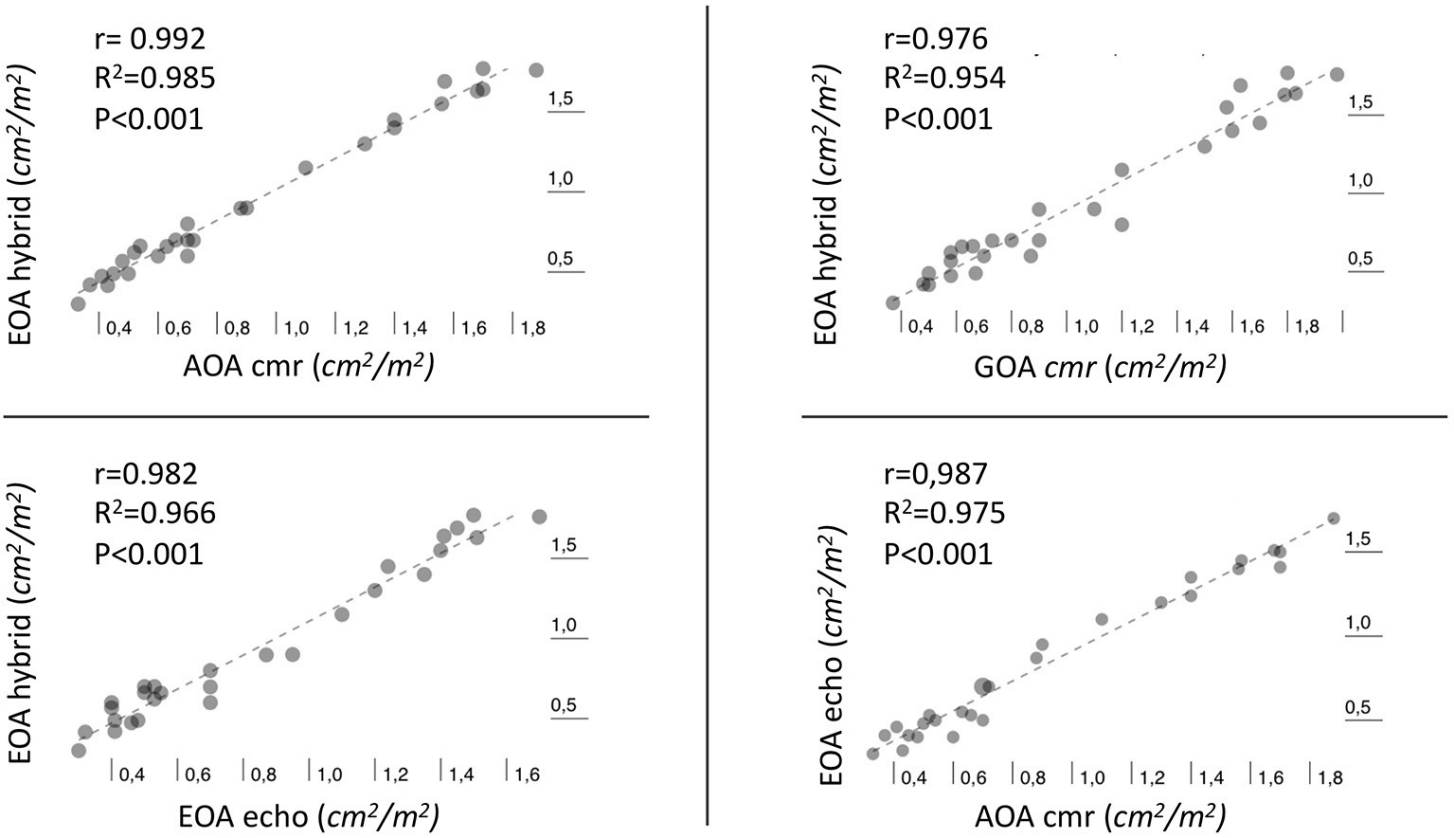
Scatter plot with best-fitting regression line illustrating the Pearson correlation (*r*) and the coefficient of determination *R*^2^ between different methods of estimation of aortic valve area.

**Figure 4:**
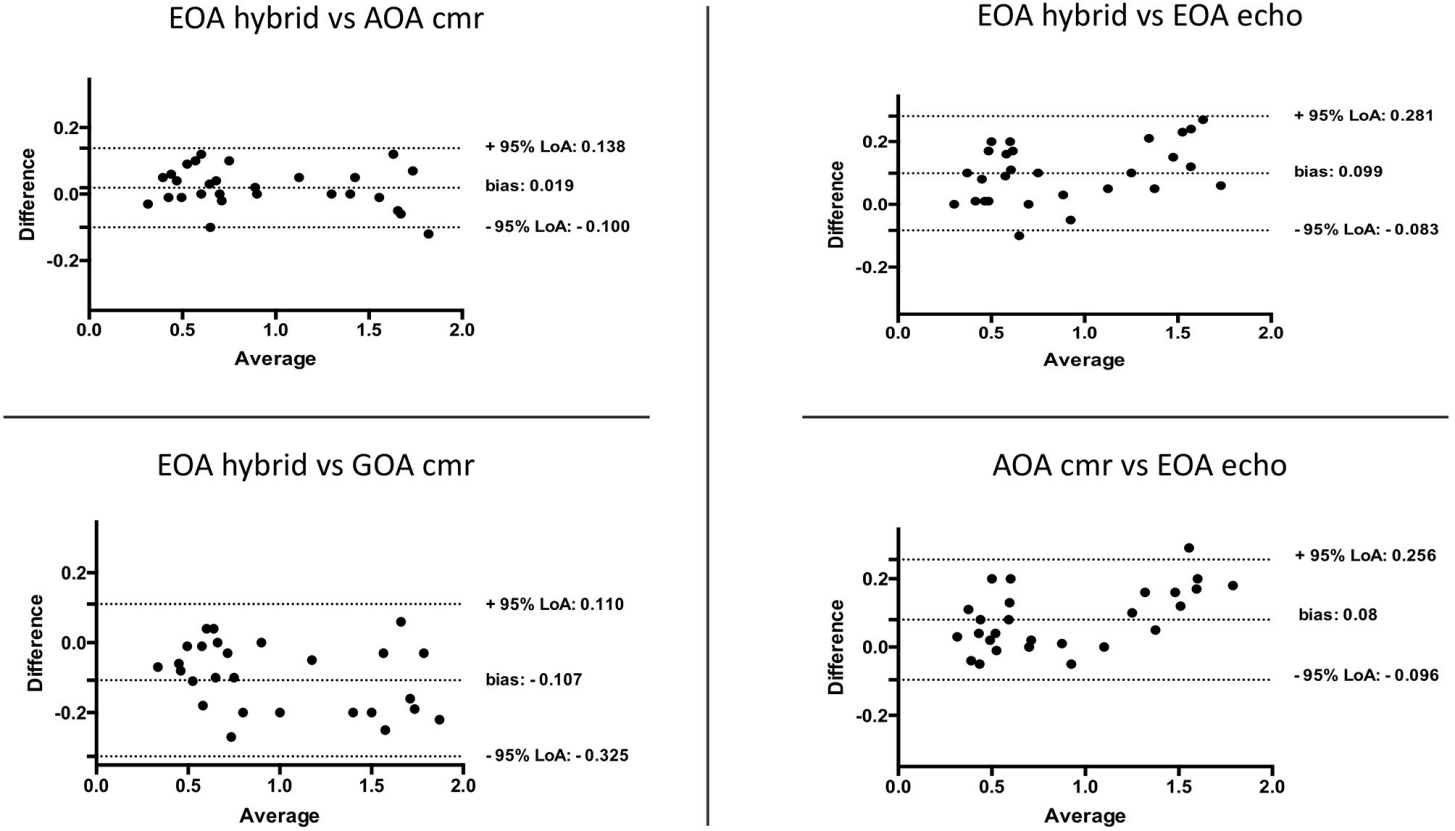
Bland–Altman plots assessing the agreement between different methods for estimation of aortic valve area.

We observed excellent pairwise positive linear correlations among AOA_cmr_, EOA_hybrid_, GOA_cmr_, and EOA_echo_ (*p* < 0.001); AOA_cmr_ had the best correlation with EOA_hybrid_ (*R*^2^ = 0.985, *p* < 0.001). There was also an excellent correlation between AOA_cmr_ and EOA_echo_ (*R*^2^ = 0.975) (Figure 3). Bland–Altman analysis (Figure 4) demonstrated a good agreement between different techniques, with the lowest bias of 0.019 for the comparison between EOA_hybrid_ and AOA_cmr_.

**Figure 5:**
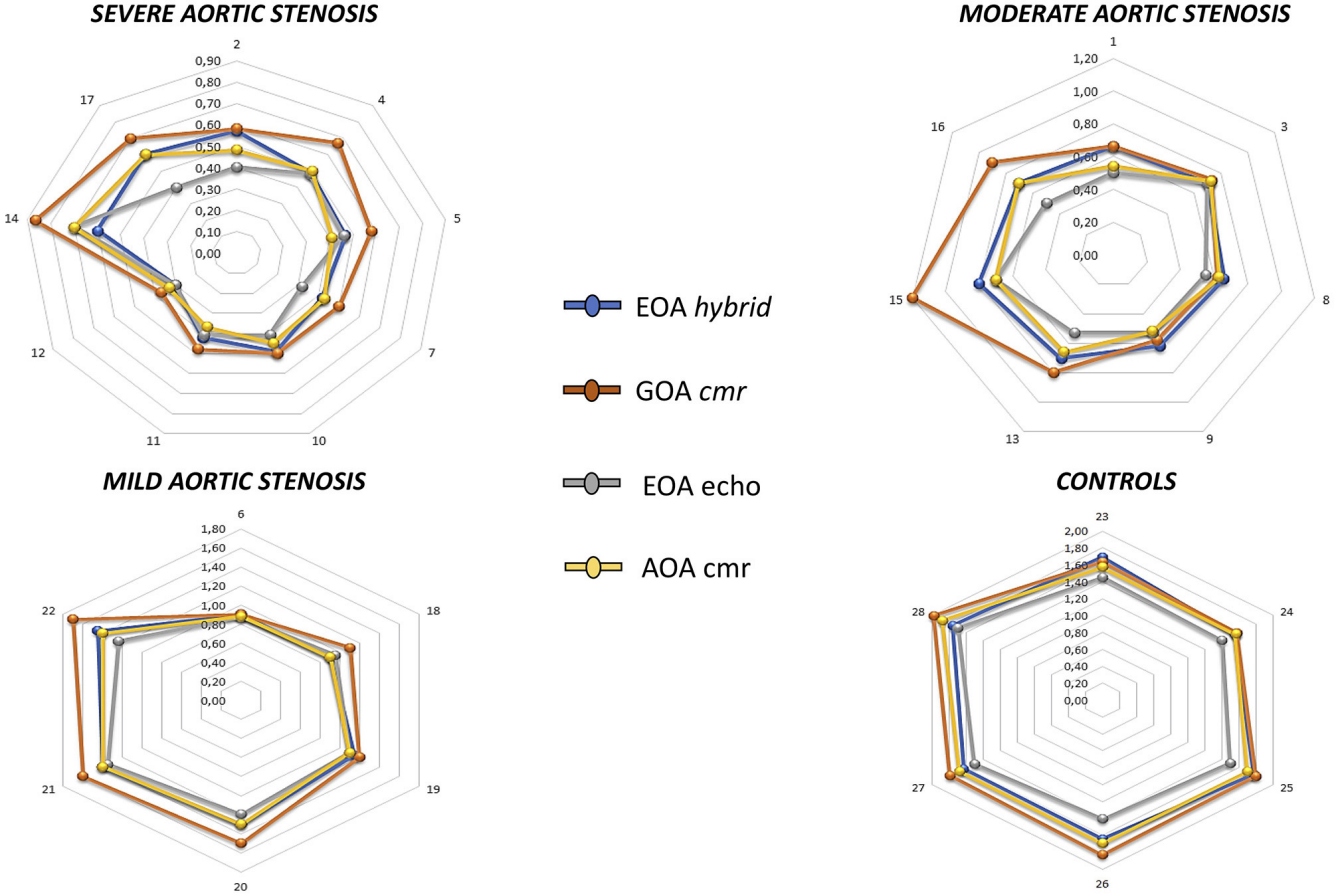
Spider diagrams showing individual aortic valve area estimates with different methods in controls and aortic stenosis patients. All measurements have been indexed to body surface area and reported as cm^2^/m^2^. AOA, aliased orifice area; EOA, effective orifice area; CMR, cardiovascular magnetic resonance; GOA, geometric orifice area; echo, echocardiography; hybrid, combined echo-CMR assessment.

Figure 5 shows spider diagrams of controls and AS patients, differentiated on the basis of the AS severity. This graphic representation allows immediate comparison of the data obtained for each individual patient. It provides a visual display confirming that GOA_cmr_ values were significantly higher than corresponding values of EOA_echo_ in all degrees of stenosis (*p* < 0.001) as well as in controls (*p* < 0.001). On the other hand, the TTE method (EOA_echo_) underestimates the valve area compared to the hybrid method (EOA_hybrid_), because of different values for LVOT area used in the continuity equation, while AOA_cmr_ values are closer to the EOA_hybrid_ values.

### Reclassification

**Figure 6:**
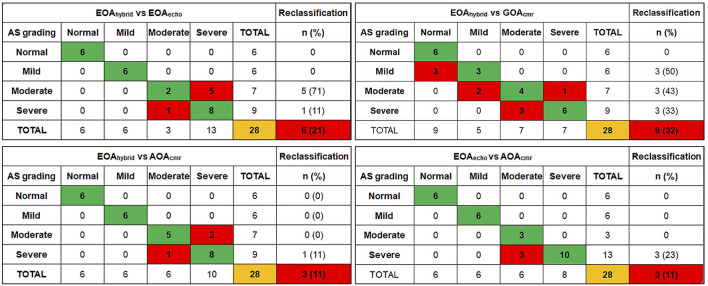
Reclassification analysis of aortic stenosis severity according to different imaging methods. This plot allows describing the number of upward or downward reclassification (red boxes) of aortic stenosis severity according to different methods.

A reclassification analysis was conducted to determine the number of patients whose AS severity was reclassified by using AOA_cmr_, GOA_cmr_, and EOA_echo_ compared with grading provided by EOA_hybrid_. Compared with EOA_hybrid_, AOA_cmr_ reclassified 3/28 (11%) subjects (Figure 6): two patients moved from the moderate to the severe category, with one patient moving from severe to moderate AS. EOA_echo_ and GOA_cmr_ reclassified, respectively, 6/28 (21%) and 9/28 (32%) subjects. When using EOA_echo_ as the reference, AOA_cmr_ underestimated AS severity in three patients (11%).

### Reproducibility

GOA_cmr_ and AOA_cmr_ yielded excellent intra-rater and inter-rater reliability both in the overall population (GOA_cmr_, absolute mean difference: 0.07 and 0.09; ICC: 0.995 and 0.991; respectively; AOA_cmr_: absolute mean difference 0.08 and 0.08; ICC: 0.997 and 0.998; respectively) and in the severe AS subgroup (GOA_cmr_, absolute mean difference: 0.08 and 0.08; ICC: 0.997 and 0.994; respectively; AOA_cmr_: absolute mean difference 0.07 and 0.08; ICC: 0.998 and 0.998; respectively).

## Discussion

In this pilot study, we described a novel CMR-based method for the measurement of the effective orifice area, which is highly accurate and reproducible in aortic stenosis patients across a range of disease severity. Direct measurement of aliased 2D *vena contracta* of the flow field through the stenotic aortic valve aims (i) to reduce sources of error associated with continuity equation calculation for assessment of EOA, and (ii) to improve diagnostic accuracy and reproducibility.

### Limitations of Echocardiography

TTE currently is the reference method for the diagnosis and quantification of AS. It is completely non-invasive and relatively simple to perform and allows an accurate diagnosis in the majority of patients (1, 4). The quantitative evaluation of AS relies on Doppler measurement of transvalvular gradients and the derivation of valve area. Both sets of metrics correlate with symptoms, need for valve intervention, and survival. TTE often underestimates LVOT area. Maes et al. showed that use of LVOT area by CMR into the continuity equation resulted in a 29% increase in the SVi and AVAi, leading to reclassification from echo-severe to CMR-moderate AS in 25% of patients (18). This is a consequence of the elliptical shape of the LVOT cross-sectional area, which is ignored by 2D TTE.

### CMR for AS Assessment

When echocardiographic parameters are discordant, inconclusive, or not accurate, CMR has proven to be a robust alternative imaging modality for grading AS severity by direct aortic valve orifice planimetry (19–22). Balanced SSFP sequences allow assessment of the anatomy and function of the AV, allowing a direct planimetry of the valve orifice (21). However, direct planimetry of the AVA can be challenging due to heavy calcifications of the tips, with image artifacts and partial volume effects. CMR also provides other relevant information including morpho-functional assessment of cardiac chambers, detection and quantification of other valvular lesions, tissue characterization, and extracardiac findings (23).

By fundamental hydrodynamic principles (the flow contraction phenomenon), the GOA is usually significantly larger than the EOA (10). This is not surprising, since direct planimetry reflects the anatomical orifice area, while the calculated EOA reflects the functional orifice area, measured after jet contraction downstream from the orifice. An *in vitro* study using stenotic bioprosthetic valves showed that an EOA of 1 cm^2^ corresponds to a GOA of 1.2 cm^2^, suggesting that a GOA measured with planimetry between 1 and 1.2 cm^2^, by either CMR imaging, computed tomography, or transoesophageal echocardiography, should not discard the possibility of severe AS.

### EOA by CMR—Conventional Approach

CMR has emerged as an alternative method for non-invasive estimation of EOA with the use of velocity-encoded CMR techniques, employing the continuity equation. CMR allows a direct measure of LVOT area avoiding its echocardiographic underestimation. However, CMR underestimates velocities compared to Doppler. This underestimation has been attributed to intravoxel dephasing, loss of signal, and lower temporal resolution than echocardiographic Doppler-based methods (10).

### Hybrid EOA

As mentioned earlier, accuracy in quantifying AS could be improved by the use of a multimodality metric, EOA_hybrid_, combining strengths and avoiding the pitfalls of both TTE and CMR. The continuity equation can be obtained by using LVOT area derived from CMR and velocities measured by Doppler TTE (11–15). Although conceptually attractive, this approach is cumbersome and not easily applicable in clinical routine, as it would require a costly sequential acquisition of two different diagnostic tests possibly under the same hemodynamic conditions.

### Aliased Orifice Area: A Novel CMR-Based Approach to AVA Measurement

To overcome the aforementioned limitations, we are reporting a new, simple CMR method for measuring EOA directly, based on phase contrast velocity mapping of the aliased orifice area. The VENC (velocity-encoded value) was intentionally low (50 cm/s) in order to enhance aliasing. The transvalvular aliased flow surface area, which we termed aliased orifice area (AOA_cmr_), is conceptually identical to the EOA. EOA is indeed equivalent to the cross-sectional area of the *vena contracta* of the transaortic flow jet. Despite the modest sample size of the study population, we obtained significant results demonstrating that AOA_cmr_ is a feasible, precise, and reproducible method to measure EOA by CMR.

We compared EOA_hybrid_, conceptually the “gold standard” for measuring EOA, with other measurement techniques (EOA_echo_, GOA_cmr_), including our new proposed index (AOA_cmr_). AOA_cmr_ proved the most accurate parameter: it had high linear correlation and excellent agreement with EOA_hybrid_, as well as a good inter- and intra-rater variability.

There are several advantages to this method: (i) single direct measurement (with fewer sources of error, and thus more precise and reproducible than the continuity equation) from a single free-breathing sequence with no need for scout sequences to set any “correct” VENC (one-fits-all approach with fixed low VENC); (ii) purely non-invasive (no contrast required); (iii) good intra- and inter-rater reproducibility; (iv) simultaneous evaluation of effective regurgitation orifice area in mixed aortic valve disease; and (v) “Doppler-like” visual inspection of the aortic valve plane (i.e., detection of peri-valvular/prosthetic leak, cusp perforation, and central or eccentric origin of the regurgitant jet).

Possible disadvantages include the following: (i) low-quality images in case of poor ECG triggering, with potentially low applicability to patients with arrhythmias; (ii) intravoxel dephasing; (iii) low temporal and spatial resolution; (iv) 2D measurement; (v) unknown dependency on flow rate and magnet field intensity; and (vi) longer velocity mapping sequence due to high number of excitations (five averages: ~2 min).

### Study Limitations

We acknowledge a few limitations that must be addressed. Firstly, the small sample size of this pilot study prevented us from including a more heterogeneous population to assess various hemodynamic subsets of AS. Secondly, we used a 2D phase contrast sequence that is sensitive to the through-plane motion of *vena contracta*. Thirdly, we used an arbitrary very low VENC value of 50 cm/s; however, the selected threshold is lower than two standard deviations below normal mean aortic valve peak velocity by CMR (i.e., 80 cm/s) (24), which makes this a reasonable cut-point to achieve aliasing at the level of the aortic valve. Fourthly, we cannot foresee the influence of field strength, gradient echo sequences, and prospective triggering on the accuracy and precision of AOA. Further studies are warranted to assess reliability and accuracy of AOA across different hemodynamic patterns of AS and different VENC values, and to explore the feasibility of 3D vena contracta area by novel highly accelerated compressed sensing 4D and 5D respiratory-motion resolved flow sequences.

## Conclusions

Aliased orifice area planimetry by low-VENC phase-contrast CMR imaging is a simple, reproducible, accurate, and precise technique for grading of AS severity. Larger studies are warranted to confirm our preliminary findings.

## Data Availability Statement

The raw data supporting the conclusions of this article will be made available by the authors, without undue reservation.

## Ethics Statement

The studies involving human participants were reviewed and approved by Comitato Etico delle Province di Chieti e Pescara e dell'Università degli Studi G. d'Annunzio di Chieti-Pescara. The patients/participants provided their written informed consent to participate in this study.

## Author Contributions

CM conceived the analysis, collected the data, and is the scientific guarantor of this publication. MK contributed to the design of the analysis and writing of the manuscript. All authors contributed to the design and implementation of the research, analysis of the results, and writing and critical revision of the manuscript.

## Conflict of Interest

The authors declare that the research was conducted in the absence of any commercial or financial relationships that could be construed as a potential conflict of interest.

## Publisher's Note

All claims expressed in this article are solely those of the authors and do not necessarily represent those of their affiliated organizations, or those of the publisher, the editors and the reviewers. Any product that may be evaluated in this article, or claim that may be made by its manufacturer, is not guaranteed or endorsed by the publisher.
